# Binned and Quality Controlled Nitrate Data from National Science Foundation Ocean Observatories Initiative Endurance Array Profilers on the Newport Hydrographic Line, 2014-2025

**DOI:** 10.1016/j.dib.2026.113381

**Published:** 2026-10-28

**Authors:** Andrew L. Scherer, Melanie R. Fewings

**Affiliations:** College of Earth, Ocean, and Atmospheric Sciences, Oregon State University, 104 CEOAS Admin. Bldg., Corvallis, OR, 97331-5503, USA

**Keywords:** California Current, Oregon, Continental shelf, Eastern boundary upwelling system

## Abstract

The northern California Current System is an oceanic eastern boundary upwelling system known for its high biological productivity. This biological productivity is driven, in large part, by the upwelling of nitrate in the summer upwelling season. Nitrate therefore serves as an important biogeochemical tracer, strongly influenced by both bottom-up physical dynamics (e.g., upwelling) and top-down biological dynamics (e.g., phytoplankton uptake). However, long-term time series of vertical profiles of in situ nitrate concentrations remain rare. The National Science Foundation (NSF) Ocean Observatories Initiative (OOI) Endurance Array has addressed that data gap by measuring in situ nitrate concentrations in spring and summer using Sea-Bird SUNA V2 instruments mounted on WET Labs Autonomous Moored Profilers (AMPs) deployed from April 2014 through September 2025. Two of these AMPs are installed at sites located on the inner-shelf (CE01, 44.662°N, 124.096°W, 25 m water depth) and the mid-shelf (CE02, 44.6372°N, 124.299°W, 81 m water depth) on the Newport Hydrographic Line (NHL) on the Oregon continental shelf. Nitrate concentration is recorded by the SUNA V2 while a collocated Sea-Bird FastCAT 49 provides conductivity, temperature, and depth (CTD) data. The nitrate concentration data were first quality-controlled by examining the underlying spectra associated with nitrate measurements, using the spectra and collocated salinity measurements to identify questionable nitrate measurements, and discarding these data. These quality-controlled nitrate data were then binned into 1 m depth bins and 1 day time bins. Finally, these binned and quality-controlled nitrate data were compared with ship-based discrete nitrate bottle samples to apply per-deployment baseline offsets and further validate the optical nitrate data from the profilers. The raw nitrate data and binned and quality-controlled nitrate data are available via Zenodo at https://doi.org/10.5281/zenodo.21478745.

Specifications Table

**Table:** 

Subject	Earth & Environmental Sciences
Specific subject area	Oceanography
Type of data	Raw, Analyzed
Data collection	Nitrate data were collected in summers from April 2014 to September 2025 by Seabird SUNA V2 instruments aboard WET Labs Autonomous Moored Profilers (AMPs). These data were accessed via the National Science Foundation (NSF) Ocean Observatories Initiative (OOI) Full Resolution Dataset catalog via the OOI Gold Copy Thematic Real-time Environmental Distributed Data Service (THREDDS) catalog. The nitrate data as acquired from the OOI Gold Copy THREDDS catalog are at the “L2” stage, i.e., the data have already been converted from the raw format on the SUNA V2 to a nitrate concentration, and a salinity and temperature correction has been applied. Conductivity, temperature, and depth (CTD) data collected from a Sea-Bird FastCAT 49 CTD are included in the dataset as well, although these variables are not the focus of this article and are included as provided by OOI for context.
Data source location	NSF Ocean Observatories Initiative Endurance Array Station, Profiler, Latitude, Longitude, Water Depth (m) CE01, CE01ISSP, 44.662°N, 124.096°W, 25 CE02, CE02SHSP, 44.6372°N, 124.299°W, 81
Data accessibility	Repository name: Zenodo Data identification number: 10.5281/zenodo.21478745 Direct URL to data: https://doi.org/10.5281/zenodo.21478745
Related research article	none

## 1 Value of the Data

• This unique, high spatial and temporal resolution, long-term dataset of vertical profiles of nitrate concentration enables new insights into physical and biogeochemical processes on the highly productive Oregon continental shelf [1]. This new nitrate dataset is available alongside decades of existing physical oceanographic observations on the Newport Hydrographic Line [2,3], supporting interdisciplinary continental shelf research [4].
• Understanding the temporal and spatial variability of nitrate is of considerable importance, as nitrate is the primary limiting macronutrient within the northern California Current System [5]. Nitrate therefore provides a link between physical and biological continental shelf processes and is often utilized when considering the effects of physical forcings on continental shelf ecosystem productivity [6,7].
• The impacts of long-term variability and climate change on nitrate (and biogeochemistry more broadly) is one of the most poorly constrained aspects in our understanding of past, present, and future changes in Eastern Boundary Upwelling Systems (EBUS) [8,9], such as the California Current System. This dataset, while not yet long enough to observe the effects of climate change, provides an important, ten-year baseline for future research on biogeochemical changes within EBUS.
• Optical nitrate data, especially when obtained from instruments mounted on long-term ocean observing platforms, can suffer from issues such as biofouling, unknown absorbers, and sensor drift [10]. The quality-control process presented here addresses these issues and, alongside comparison with ship-based bottle samples, provides a validated nitrate dataset for use on the Oregon shelf, with methods that are potentially applicable to other optical nitrate datasets.

## 2 Background

The primary motivation for providing this dataset is to make available a curated, long-term, analysis ready oceanic nitrate concentration dataset. Long-term, high-resolution nitrate measurements are rare in EBUS globally, resulting in poor constraints on past, present, and future changes in continental shelf biogeochemistry [8,9]. The OOI Endurance Array has made an important contribution toward closing this data gap by providing over ten years of high-resolution, vertically profiling nitrate measurements on the Oregon continental shelf [11], located within the California Current System EBUS. However, the OOI Endurance Array provides these in situ nitrate observations un-binned and without outliers removed or biases corrected [10].

The primary contributions of this work are to provide a new algorithm that uses the underlying spectra to determine the validity of the individual optical nitrate measurements, to bin the data in regular depth-time bins, and to validate and align these measurements with discrete, ship-based nitrate bottle sample measurements. These data are then made available in curated, Climate and Forecast (CF) metadata compliant, analysis-ready netCDF files for use in future research.

## 3 Data Description

Binned and quality-controlled nitrate concentration along with collocated Practical Salinity, Absolute Salinity, in situ temperature, Conservative Temperature, and potential density anomaly are available via Zenodo at https://doi.org/10.5281/zenodo.21478745 [12]. The dataset consists of two directories, one for each OOI profiler described in this article: *CE01ISSP* [13] and *CE02SHSP* [14] (Fig. 1). Each directory contains one CF metadata compliant netCDF file that contains the binned, quality controlled, and baseline shifted nitrate concentrations with the name *PROFILER_nitrate_binned_qced_baseline_subtracted_YYYY-MM-DD_YYYY-MM-DD.nc*, where the profiler name and the start and end dates of the data are included in the file name. Another netCDF file is included that contains the binned and quality-controlled nitrate concentrations without the baseline shift applied, in case users would prefer to apply a different baseline subtraction, with the name *PROFILER_nitrate_binned_qced_YYYY-MM-DD_YYYY-MM-DD.nc* and the same format as previously. The raw data files for each deployment are available from the OOI Gold Copy THREDDS catalog, accessible online at the links found in [13] and [14] for profilers CE01ISSP and CE02SHSP, respectively. These raw data files are also provided, without modification, in the *raw/* subdirectory with file names given by *PROFILER*_*depXX_YYYY-MM-DD_YYYY-MM-DD.nc*, where *XX* refers to the deployment number and with other formatters as previously described.

**Fig. 1:**
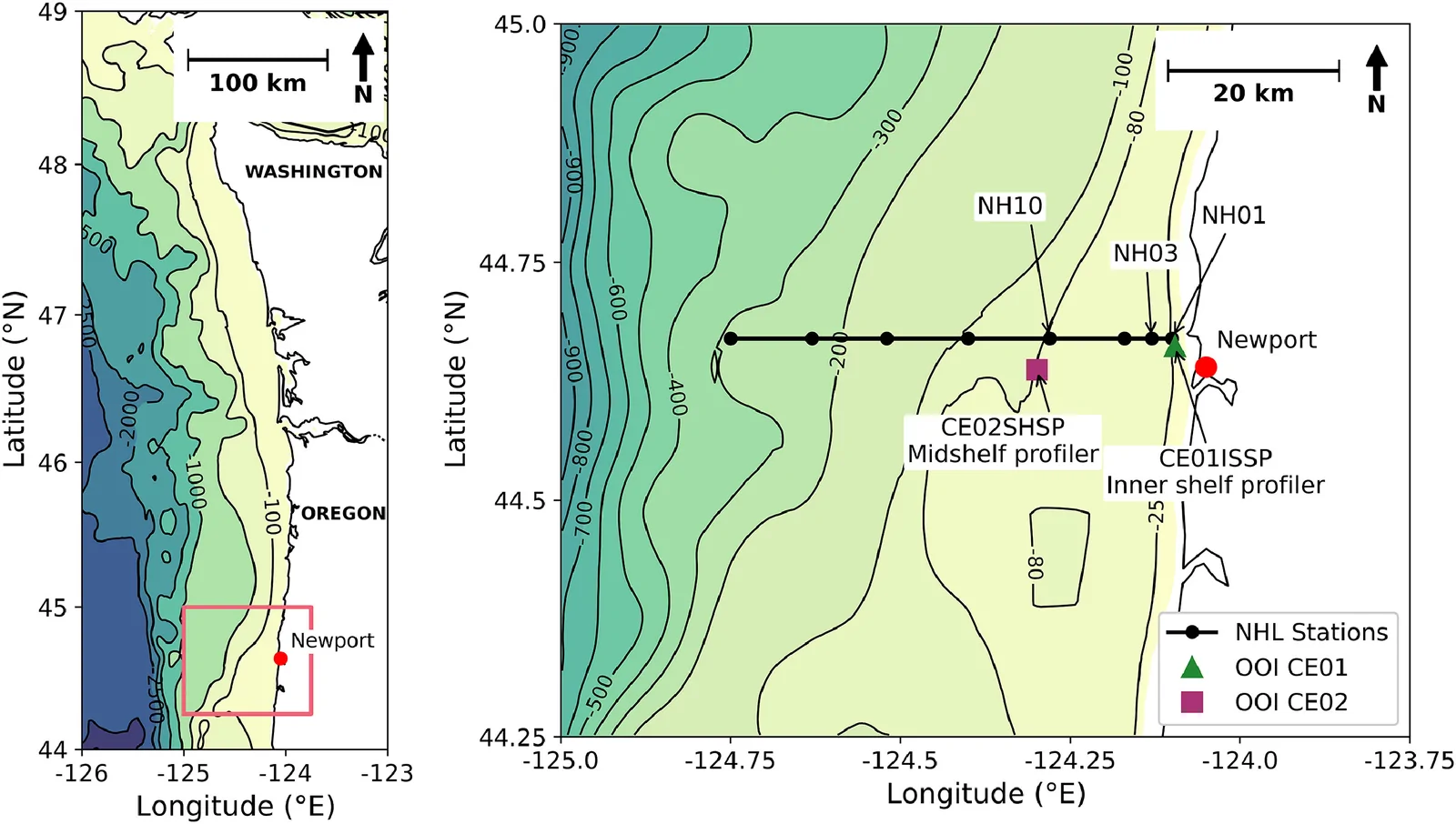
The data collection region off the northwest U.S. and relevant observational assets. The red box in the left panel indicates the area covered by the detailed map in the right panel. Isobaths are labeled in meters. The two NSF OOI profilers that collect nitrate data are CE01ISSP and CE02SHSP. Collocated bottle samples used for validation come from both OOI cruises that sampled at OOI sites CE01 and CE02 and from NHL shipboard surveys taken at the NH01 and NH03 stations (used to validate CE01ISSP data) and at the NH10 station (used to validate CE02SHSP data). The red dot in both panels indicates Newport, OR, USA.

Each profiler directory also includes two netCDF files that contain the bottle sample data used to validate the in situ nitrate concentrations at that site. At each site, bottle samples come from either National Oceanic and Atmospheric Administration (NOAA) NHL sampling cruises or OOI cruises [15] (Fig. 1) and include both the discrete nitrate measurement and collocated CTD data. Within the *CE01ISSP* directory, discrete nitrate samples and collocated CTD data sampled at NHL stations NH01 and NH03 stations (Fig. 1) are in the file *nh01_nh03_nitrate_bottles.nc* and the same data sampled at the OOI site CE01 are in the file *ce01_nitrate_bottles.nc*. Within the *CE02SHSP* directory, discrete nitrate samples and collocated CTD data sampled at the NH10 station (Fig. 1) are in the file *nh10_nitrate_bottles.nc* and the same data sampled at the CE02 site are in the file *ce02_nitrate_bottles.nc*.

Finally, the full quality control, binning, validation, and offsetting algorithm described in this article is available alongside the data in another top-level directory with the name *ooi-profiler-nitrate-retriever* and is also available in a GitHub repository at https://github.com/andrew-s28/ooi-profiler-nitrate-retriever. Within the directory *ooi-profiler-nitrate-retriever* is a Python script which can be used for applying the algorithms described herein to the most up-to-date data available on the OOI Gold Copy THREDDS catalog. Also included in that directory is an example notebook which walks through each step of the algorithm alongside the analysis code and visualization.

## 4 Experimental Design, Materials and Methods

### 4.1 Data collection

The Ocean Observatories Initiative (OOI) Endurance Array deploys WET Labs Autonomous Moored Profilers (AMPs) nominally for three-month deployments in the spring and summer (April-September), as rough seas in the winter mean that the profilers must be removed. The AMPs profile upward and typically take six hours to complete a profile, meaning about four profiles are completed per day. Profiles ideally span the entire depth range, from about a meter above the seafloor to the surface, but the profiler often cannot complete full profiles due to wave conditions or other hazards, so sampling frequency is not always consistent across depths. One AMP is deployed at each site, profiler CE01ISSP at site CE01 (44.662°N, 124.096°W, 25 m water depth) and profiler CE02SHSP at site CE02 (44.6372°N, 124.299°W, 81 m water depth) (Fig. 1).

A Sea-Bird SUNA V2 is mounted on the frame of each AMP roughly 0.5 m below a Sea-Bird FastCAT 49 CTD. A brush is mounted within the sampling volume of the SUNA V2 that rotates over to clean the optical sensors once per profile. The SUNA V2 records the intensity of UV light over a wavelength range of 190 nm to 370 nm passed through a sample path length of 1 cm. The SUNA V2 was initially set up to record a light spectrum approximately once every 12 s (similar to the “AUV SUNA” setup described in Table 3.2 of [10]) to prevent the lamp from overheating. This results in a vertical resolution of approximately 3 m, depending on the upward velocity of the profiler (typically around 0.25 m/s). This changed in 2019, when it was determined that lamp heating was not an issue during profiles, and thus the measurement frequency was changed to about once every second, resulting in sub-meter vertical resolution thereafter.

The “L0” absorption data obtained by the SUNA V2 instrument is processed by OOI, with the recorded spectra processed through an algorithm that corrects for the effects of other absorbers, including the temperature-dependent absorption of bromide [10]. Each corrected spectrum is then used to compute a salinity and temperature corrected nitrate concentration based on a spectral deconvolution technique applied to the wavelength range from 217 nm to 240 nm [10,16]. The salinity and temperature corrected nitrate data are then provided as “L2” OOI data products. Both the L0 absorption spectra and the L2 salinity and temperature corrected nitrate concentration were retrieved from the OOI Gold Copy THREDDS data server as described in the Data Description section above.

### 4.2 Data processing

The in situ temperature, Practical Salinity, and pressure are provided by OOI from a collocated FastCAT 49 CTD; we use these CTD data as provided by OOI without modification. We then use the CTD data to calculate Absolute Salinity, Conservative Temperature, and potential density anomaly ($\sigma_{\theta }$) using the Gibbs Seawater Oceanographic Toolbox [17]. These derived variables are provided alongside the nitrate data in each profiler dataset.

Prior to binning, we apply a quality control algorithm to the L2 temperature and salinity corrected nitrate data that removes outliers based on the L0 absorbance spectrum of each nitrate measurement and the collocated salinity measurements. We apply this algorithm to every observation independently:

1) If the OOI Quality Assurance of Real Time Ocean Data (QARTOD) flag indicates bad data (a value of 4), remove this observation.
2) If the practical salinity is less than 28 PSU, remove this observation. The nitrate salinity and temperature correction algorithm was not tested for values lower than this cutoff and often produces outlier nitrate measurements as a result [16].
3) If there are any “not a number” (NaN) or negative values in the spectrum, remove this observation. This means the instrument malfunctioned in some way or that the optical path was severely impacted (e.g., by dense particulate matter).
4) Fit the spectrum between wavelengths λ of 360 nm and 380 nm with a linear least squares regression of the form $\hat{y}=\beta_{1}\lambda +\;\beta_{0}+\varepsilon$, with regression coefficients $\beta_{1}$ and $\beta_{0}$ to be determined by the fit and ε the residual of the fit. This wavelength range is far from the absorption peaks of nitrate or seawater [16], so the major absorber is expected to be chromophoric dissolved organic matter (CDOM). CDOM should have an absorption spectrum that is approximately linear, at least over small wavelength ranges [18]. A linear dependence on wavelength is explicitly accounted for in the salinity and temperature correction algorithm [16], but if the background spectrum is not approximately linear, then the accuracy of the calculated nitrate is suspect. Thus, we remove any observation associated with spectral fits where the root mean square error is greater than 0.02 counts or that fail to converge.
5) Finally, the mean and standard deviation of the slopes of the linear fits from the previous step are calculated for all the "good" data as determined by steps 1–4. Any observation associated with a best-fit spectrum with a slope more than three standard deviations away from the mean slope over all observations is removed. A strong deviation from the mean is indicative of an unknown absorber present in the optical path, which makes the accuracy of the measurement suspect.

This quality control algorithm removes from 0.01% to 5% of data from most deployments (Table 1). Some deployments have >50% of the data removed by this algorithm, indicating that something may have gone wrong with the entire deployment. Deployment 20 of CE01ISSP, which spanned from 07/15/2022 to 09/30/2022, had nearly 60% of the data removed. Deployment 02 of CE02SHSP, which spanned from 03/18/2015 to 04/15/2015, had all data removed. Both deployments were removed entirely from the final dataset, as it is assumed that there was something wrong with the deployment or instrument configuration itself. Any deployments with fewer than ten total profiles were also removed, since this means something likely went wrong with this installation of the profiler for this deployment and the profiler had to be recovered early. This removed deployments 11 and 16 of profiler CE01ISSP and deployments 26, 29, 30, and 35 of CE02SHSP. The results of this algorithm on the remaining deployments of CE01ISSP and CE02SHSP are shown in Table 1. The effect of each of these steps on the data from deployment 17 of profilers CE01ISSP and CE02SHSP is shown in Figs. 2 and 3, respectively.

**Table 1:** The number of observations removed in each step of the quality control algorithm for profilers CE01ISSP and CE02SHSP. The total number of observations N before the quality control algorithm is shown alongside the profiler name in the first row.

	**CE01ISSP** (*N* *=* *123,009*)	**CE02SHSP** (*N* *=* *351,733*)
Removed in Step 1	458	446
Removed in Step 2	40	29
Removed in Step 3	7079	2800
Removed in Step 4	45	0
Removed in Step 5	798	953

**Fig. 2:**
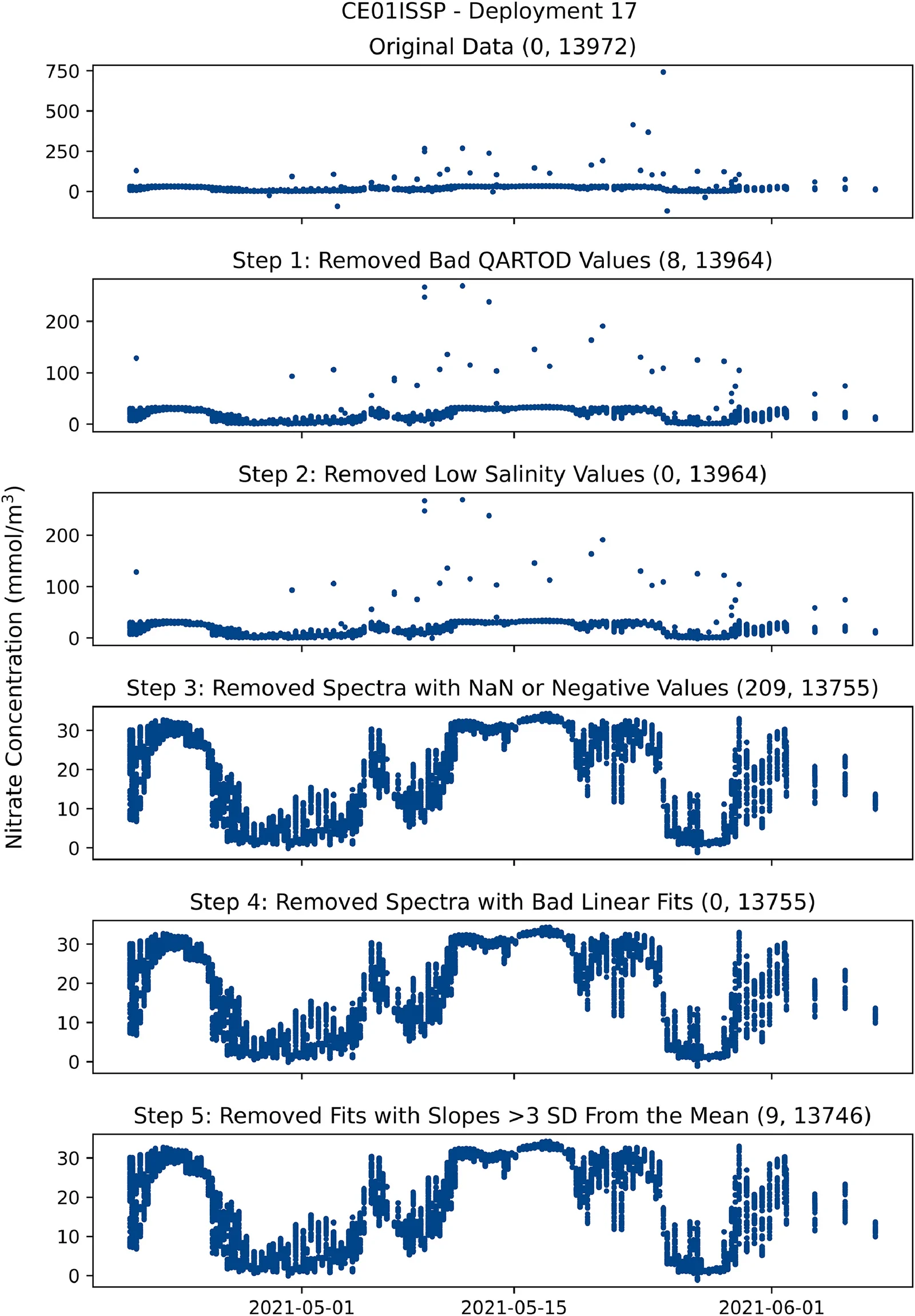
Nitrate concentration from deployment 17 of profiler CE01ISSP after each step of the quality control algorithm. The numbers in the parentheses in the title of each plot denote the observations removed in each processing step and the total observations remaining after that processing step, respectively.

**Fig. 3:**
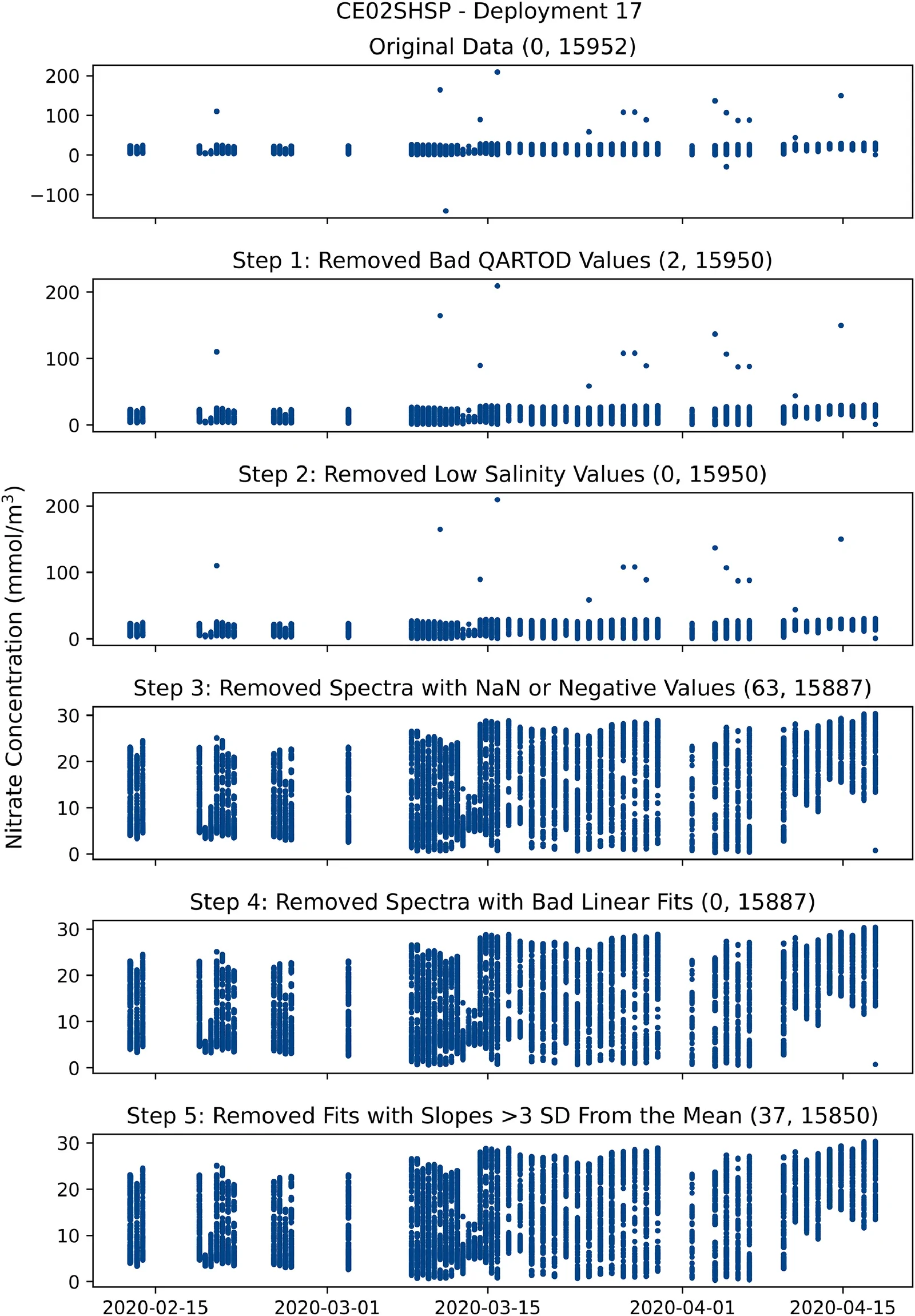
Same as Fig. 2 but for deployment 17 of profiler CE02SHSP.

Most of the discarded measurements are removed in step 1, based on the OOI QARTOD flag, step 3, based on the invalid measurements within each spectrum, and step 5, based on the spectral fits (Table 1). The QARTOD flag is based on a gross range test compared to a climatology of nitrate at each profiler. While we apply this step to be consistent with best practices when working with OOI biogeochemical datasets [10], all of the data points removed by the QARTOD flag would have been removed in later steps if the QARTOD flag step were not included. This indicates that the 5-step algorithm is successful at identifying bad data flagged by the QARTOD gross range test but goes further by identifying additional bad measurements that still fall within the QARTOD climatology range.

Following the application of the 5-step quality control algorithm, we binned the dataset, including both the quality-controlled nitrate data and the CTD data, into 1 m depth bins centered every 1 m from the surface to the bottom (26 m at CE01ISSP and 81 m at CE02SHSP), such that the edges of bins are at half-integer depths (bins are from 0.5–1.5 m, 1.5–2.5 m, etc.). To calculate the depth of the nitrate measurements, the depth measured by the CTD was offset by 0.5 m because the SUNA V2 instrument was mounted approximately 0.5 m below the CTD on the AMPs. Due to the approximately 3 m vertical resolution of the profiler data prior to 2019, some bins in this time period are empty (values in empty bins are set to NaN). Data both before and after 2019 are binned into the same 1 m depth bins without any vertical interpolation to allow users to handle these gaps according to their preferred method. The data after quality control and binning is available in the *PROFILER_nitrate_binned_qced_YYYY-MM-DD_YYYY-MM-DD*.nc file in each respective profiler directory.

### 4.3 Verification with ship-based bottle samples and application of baseline shift

Once the quality control and binning are complete, the final step in the process is to validate the optical nitrate data from the SUNA V2 against collocated, ship-based bottle samples in the region. These bottle samples come from two main sources: the OOI mooring turn-around cruises (bottle samples are taken when moorings are replaced, but this often does not align with when the profilers are replaced) and NOAA-funded broadscale surveys along the Newport Hydrographic Line (NHL). On the NHL surveys, nitrate bottle samples are taken at stations NH01 (which coincides with site CE01), NH03 (44.652°N, 123.130°W in 48 m water depth, 2 nm offshore of CE01) (Fig. 1, left panel), and NH10 (which coincides with site CE02) (Fig. 1).

While it would be ideal to have these bottle samples taken during times when the profiler nitrate sensor is actively sampling, this is often not the case. Out of 511 available bottle samples at NH01, NH03, and CE01, only 103 overlapped with profiles within one day. Out of 308 available bottle samples at NH10 and CE02, only 57 overlapped with profiles within one day. These near-coincident samples are not distributed evenly in time either; out of 35 deployments of the CE02SHSP profiler, bottle samples overlap with profiler deployments during only 20 deployments. This prevents the use of bottle samples as a direct comparison to the optical nitrate data for many deployments, since the nitrate concentrations vary significantly day-to-day (Figs. 2, 3).

Despite these complications, which prevent verifying every deployment with bottle samples, we determined that it is feasible to verify the optical data in a mean sense. There is a consistent relationship between nitrate concentration and potential density from the bottle samples at both sites, similar to what has been observed in other studies [19] (Figs. 4a and 5a).

**Fig. 4:**
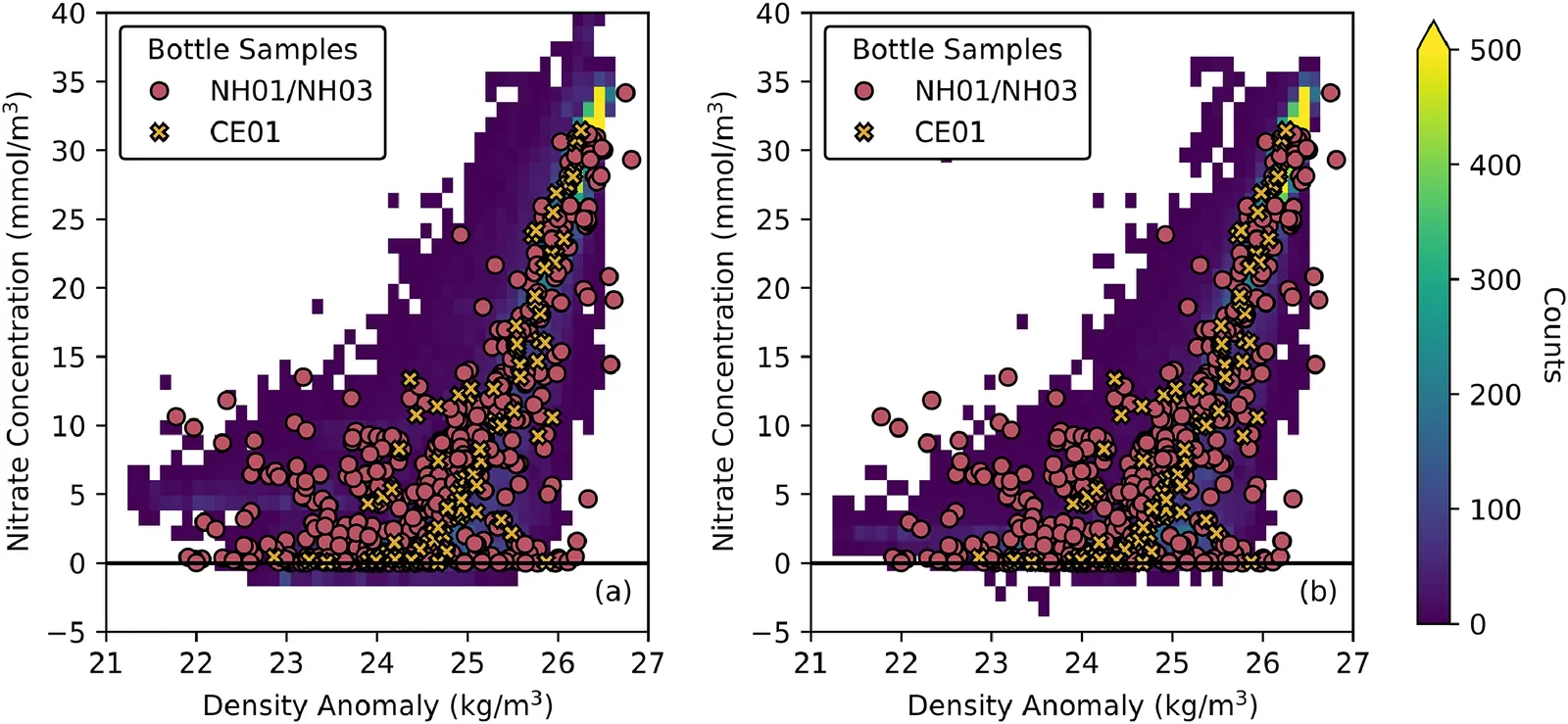
Nitrate plotted versus density for (colored background distribution) the quality-controlled nitrate data from profiler CE01ISSP and (circles and ‘x’ symbols) the ship-based bottle samples. (a) The nitrate-density relationship before a per-deployment baseline shift has been applied. (b) The nitrate-density relationship after a per-deployment baseline shift has been applied.

**Fig. 5:**
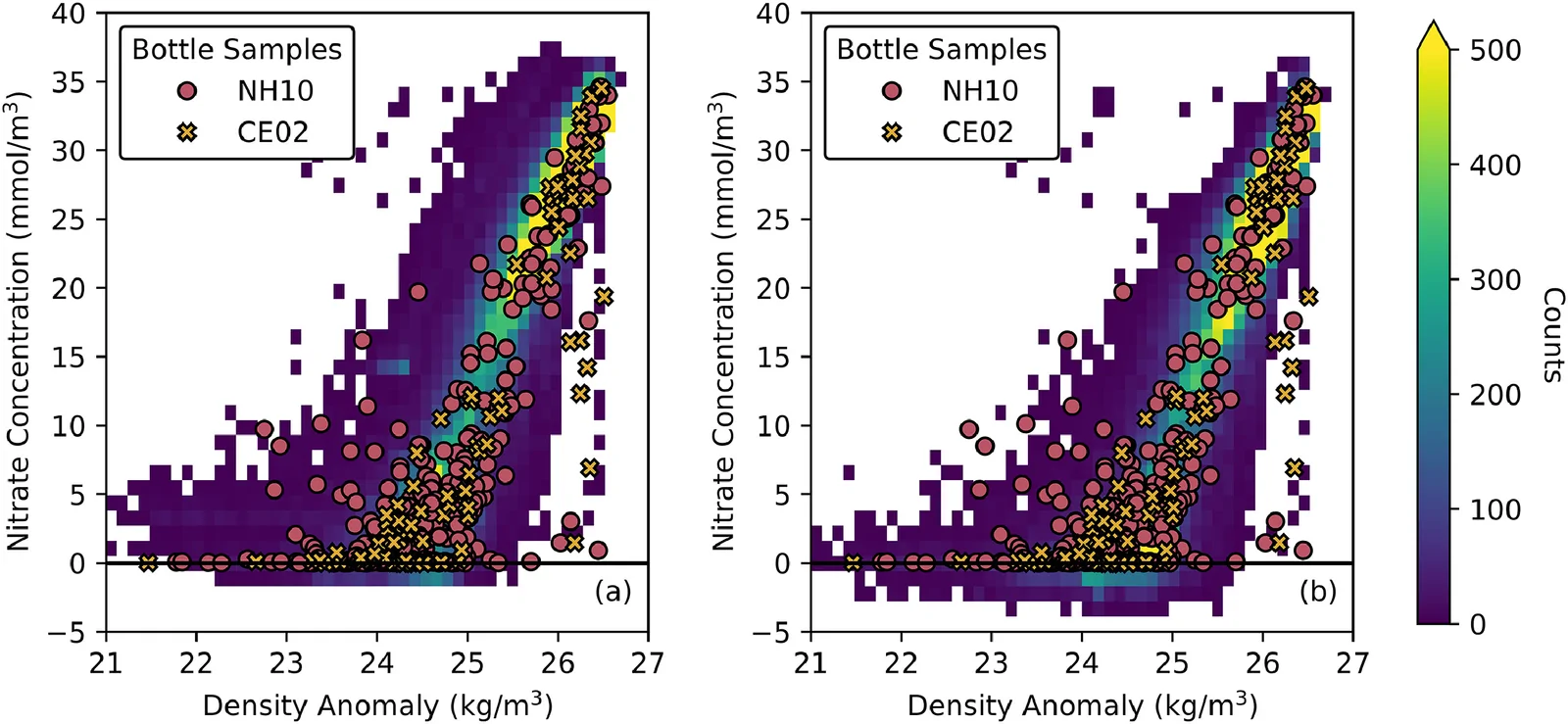
Same as Fig. 4, but for the CEO2 site and station NH10.

The profiler nitrate measurements at both sites were then observed to be offset in several deployments relative to the nitrate-density relationship displayed by the bottle samples. One way to deal with an offset is to assume that the near surface nitrate will have a near-zero median during any individual deployment [10]. This works well for the CE02SHSP profiler at site CE02, where there is relatively strong stratification with depth and nitrate is most often near zero at the surface. Therefore, at CE02SHSP, for each deployment we find the median nitrate concentration in the upper 5 m of the water column and apply a baseline offset such that this value is zero, following [10]. However, at the inner-shelf CE01ISSP profiler, the water column is more weakly stratified [20] and the surface nitrate is often non-zero, so this methodology cannot be applied there. Therefore, at CE01ISSP, for each deployment we apply a per-deployment baseline shift that is estimated to the nearest 3 mmol m^−3^. Baseline shifts determined via both methods are typically within ±3 mmol m^−3^ of the unshifted data (similar to the instrument precision). Deployments 01 and 06 were the only deployments which required a larger baseline shift of 9 mmol m^−3^ and 10 mmol m^−3^, respectively. Applying these baseline shifts brings the nitrate measurements for each deployment in line with the nitrate-density relationship identified via bottle samples. An example of this procedure for deployment 01 at CE01ISSP is shown in Fig. 6. The profiler data resulting from these baseline shifts applied to all deployments are shown compared to the bottle samples in Figs. 4b and 5b.

**Fig. 6:**
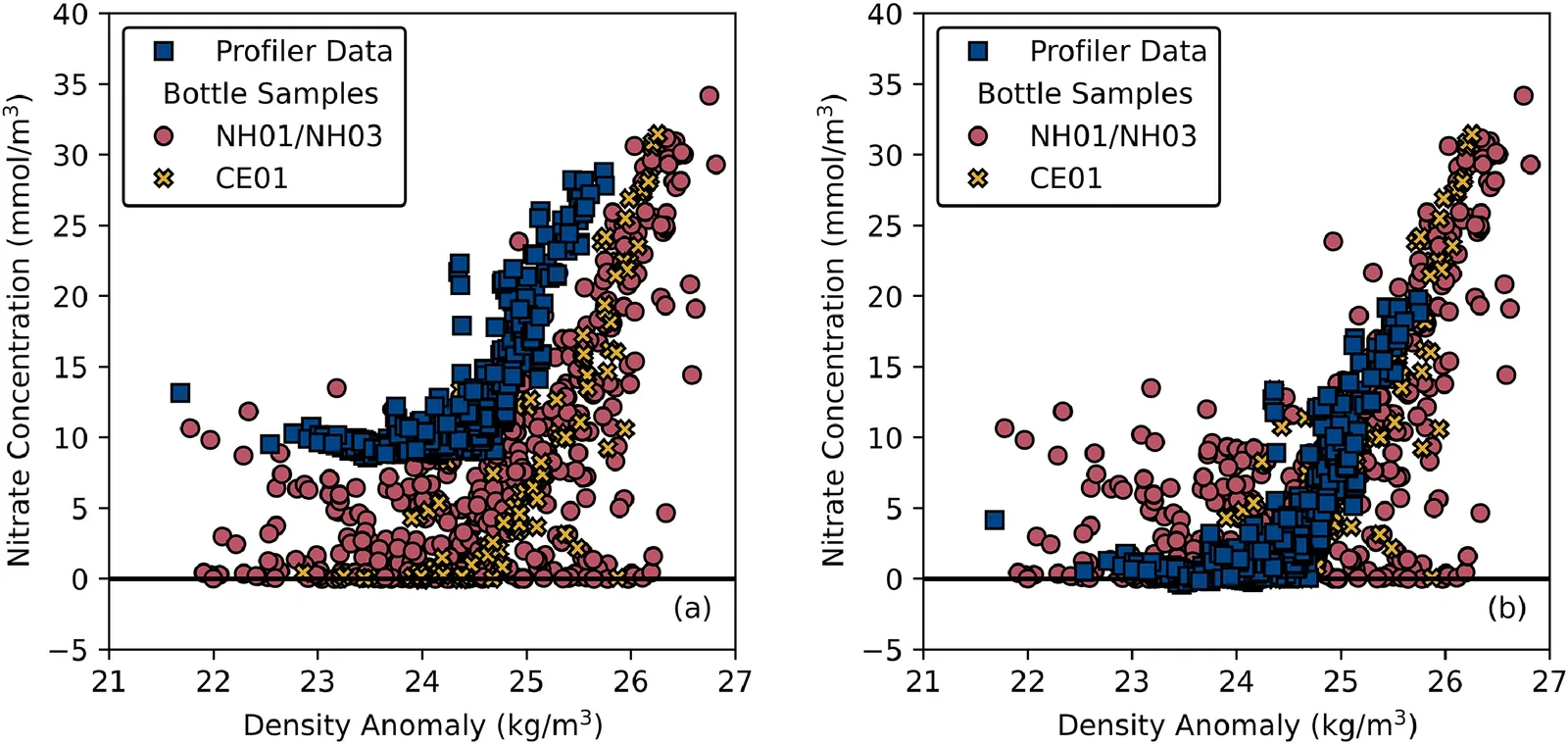
The same as in Figs. 4 and 5 but for (blue squares) only deployment 01 of the CE01ISSP profiler.

The alignment of the nitrate-density relationship from the profiler data with that found from the bottle samples also aligns the profiles of nitrate with the profiles from bottle samples for the handful of cases where they are both available within one day. However, since the baseline shifts were determined by considering each profiler deployment as a whole, not by considering individual profiles, we caution the use of any individual profile within this dataset, instead intending this dataset to be for investigating nitrate variability on timescales longer than one day.

## Limitations

One limitation of the data is that individual nitrate profiles cannot be fully validated against bottle samples due to the limited number of coincident bottle samples. However, the nitrate-density relationships with each deployment (Figs. 4 and 5) and the monthly mean nitrate profiles (not shown) from the quality-controlled and baseline shifted profiler data align well with the available bottle samples. There is also no profiler data from the winter months due to the rough sea state, limiting this dataset to the spring and summer from April to September.

## CRediT Author Statement

**Andrew L. Scherer:** Conceptualization, Data curation, Formal analysis, Methodology, Software, Visualization, Writing – original draft, Writing – review & editing. **Melanie R. Fewings:** Funding acquisition, Supervision, Writing – review & editing.

## Ethics Statement

The authors follow the ethical requirements for publication in Data in Brief and the datasets described here involved no human subjects, animal experiments or social media platforms.
